# Anatomical organization and neural pathways of the ovarian plexus nerve in rats

**DOI:** 10.1186/s13048-017-0311-x

**Published:** 2017-03-14

**Authors:** César F. Pastelín, Nora H. Rosas, Leticia Morales-Ledesma, Rosa Linares, Roberto Domínguez, Carolina Morán

**Affiliations:** 1 Facultad de Medicina Veterinaria y Zootecnia, Benemérita Universidad Autónoma de Puebla, Puebla, Mexico; 2 Departamento de Biología y Toxicología de la Reproducción, Instituto de Ciencias, Benemérita Universidad Autónoma de Puebla, Puebla, México; 3 Centro de Química, Instituto de Ciencias, Benemérita Universidad Autónoma de Puebla, Puebla, Mexico; 4 Unidad de Investigación en Biología de la Reproducción, FES Zaragoza, Universidad Nacional Autónoma de México, Ciudad de México, México

**Keywords:** Ovary, Ovarian plexus nerve, Prevertebral ganglia, Neural pathways

## Abstract

**Background:**

In this work, a detailed anatomical description of the ovarian plexus nerve (OPN) in rats is presented. The distribution of the OPN was analyzed by gross anatomy; the features of the superior mesenteric ganglion (SMG) were determined by histological studies; and the localization of the postganglionic neurons innervating the ovary were identified with retrograde tracer.

We studied 19 adult cyclic rats of the CIIZ-V strain.

**Results:**

We found that the right OPN originates from the celiac ganglion, the lumbar ganglion of the sympathetic trunk (LGST) and the SMG. The left OPN originates from the LGST and the anastomotic branch from the splanchnic nerve. The SMG was attached to the inferior vena cava containing sympathetic neurons that innervate the right ovary through the OPN, and which is anatomically single. When the tracer was injected into the right ovary, only the SMG showed positive neurons, while when the tracer was injected into the left ovary, labeled postganglionic neurons were observed in the LGST.

**Conclusions:**

This is the first time that it is reported that the SMG is attached to the inferior vena cava and it is directly related to the right ovary. The neural pathways and sympathetic ganglia involved in the communication between the ovaries and the preganglionic neurons are different in the left and right side.

## Background

In the rats, the autonomic nerves arrive to the ovary by two pathways: the superior ovarian nerve (SON) in the suspensory ligament and the OPN which runs jointly to the ovarian artery and vein [1–3]. The OPN innervates the ovary, bursa, oviduct [3], and cranial uterine horns [1]; the axons enter through the hilum [4–8] and innervate the medullary and cortical region of the ovary. The OPN is mostly composed by sympathetic and sensory fibers, with a small portion of parasympathetic fibers [4, 9, 10]. The preganglionic sympathetic fibers emerge from the spinal cord at the T9 and T10 segments [11] and the perikarya of the postganglionic sympathetic fibers are located in the paravertebral and the prevertebral ganglia [1, 6]. The sensory neurons were located in lower thoracic and upper lumbar dorsal root ganglia (DRG). The parasympathetic fibers derive from the vagus nerve [12, 13].

Houdeau et al. [6] reported clear evidence showing that some fibers of the OPN come from the suprarenal ganglion (SG). Some authors describe that the OPN originates from the prevertebral celiac-superior mesenteric ganglia (CSMG) [7, 14–17]; however, different researches [1, 5, 7, 18] sustain that the CSMG is a unilateral complex. Other authors agree that the CSMG is a bilateral one, comprising paired suprarenal, left and right celiac, superior and inferior mesenteric ganglia [17, 19]. Baljet and Drukker [2] showed that the fibers innervating the ovaries arise mainly from the celiac plexus (left and right suprarenal ganglia, left and right celiac ganglia, aorticorenal ganglion, and bundles of nerves fibers between them). Some studies found a small ganglion located near the origin of the renal and ovarian artery, which was not given a name [15, 16, 19]. Recent studies have shown that the communication between the ovaries and the prevertebral ganglia is asymmetrical [19].

The aim of the present study was to make a detailed anatomical description of the OPN in rats by using gross anatomy, histological, and retrograde tracer methods; emphasizing in the neural circuit that communicates the ovaries to the prevertebral ganglia between the left and the right side.

## Methods

### Animals and experimental design

The experiment protocol numbered VIEP/0118/2014 to use rats was approved by the Care and Use of Laboratory Animals Committee at Benemérita Universidad Autónoma de Puebla. Technical specifications related to productions, Care and Use of Animals are Specified in the Guidelines of the Mexican Council on Laboratory Animals Care (NOM-062-Z00-1999).

Nineteen 3-month-old virgin rats of the CIIZ-V strain (250–350 g body weight) were studied. The rats were maintained on a 12/12-light/dark cycle with food (LabDiet 500, Rodent diet) and water provided ad libitum. Estrous cycles were monitored by daily vaginal smears. Only rats showing at least two consecutive 4-day cycles were used in the experiment. Rats were randomly distributed to one of the following experiments: gross anatomy (*n* = 8), histological processing (*n* = 3), and retrograde-neuron tracer (*n* = 8). The surgeries were performed under anesthesia between 9:00 and 10:00 h on diestrus. The diestrus period in the rat, has the longest duration of the estrous cycle, besides the neuronal activity is the most stable [19]. All rats were sacrificed during the diestrus day a cycle after with an overdose of sodium pentobarbital (60 mg/kg, i.p. Anestesal, Smith Kline, Mexico City, Mexico).

### Gross anatomy

The rats were anesthetized with an intraperitoneal injection of urethane (ethyl carbamate, 1.2 g/kg; Sigma-Aldrich, Toluca, Mexico). For exposure of the OPN, a 3 cm paramedian skin incision was made. The OPN was dissected and the SMG was located attached to the inferior vena cava. Drawings were made using a stereoscopic microscope (Carl Zeiss Stemi 2000C, USA). The digital photographs were taken and managed in Imagen Pro Plus, version 6.3 for Windows (Media Cybernetics, Inc.).

### Histologic processing

Three rats were sacrificed with sodium pentobarbital. The skin and the abdominal muscle were cut. Using a stereoscopic microscope, the superior mesenteric ganglion (SMG) was located attached to the inferior vena cava, and it was dissected. The tissue was placed in Böuin Duboscq’s fixative solution (glacial acetic acid, formalin, and picric acid) for 12 h. The fixed tissues were rinsed, dehydrated in graded ethanol, and embedded in paraffin. Paraffin sections at 7-μm thickness were cut with a microtome (Leica RM2125RT, Germany) and collected on gelatin-coated slides. The sections were deparaffinized in xylene, rehydrated through graded ethanol, and stained with Nissl stain (used for the detection of Nissl bodies in the cytoplasm of neuron). All slides were subjected to histological analysis.

### Tracing postganglionic neurons

Eight rats in diestrus were anesthetized with an intraperitoneal injection of ketamine (90 mg/kg) and xylazine (15 mg/kg, i.p.). A unilateral incision was made 3 cm below the last rib, affecting the skin, muscle and peritoneum. Under a stereoscopic microscope, the left or right ovarian bursa was injected with 5 μl of True Blue (TB; Sigma, St. Louis, Missouri, USA), which was diluted at 1%, of distilled water; This procedure was previously reported by Lawrence and Burden [1] where they made an immersion of the cut superior ovarian nerve in the TB solution. In this study we injected the TB into the ovarian bursa [19, 20]. To prevent a leakage of the tracer, the needle was kept in the bursa for 5 min after injection. The ovary was carefully cleaned, dried, and returned to the abdominal cavity. The rats were kept in a warm chamber until their recovery from anesthesia, and they were provided with antibiotics and analgesia. After that, they were returned to their bioterio.

Four days later, the rats were anesthetized, sacrificed by transcardial perfusion with 200 mL of cold saline solution, followed by 200 mL of fixative solution (4% paraformaldehyde in phosphate buffer at pH 7.3). After perfusion the right SMG, including lumbar ganglion of the sympathetic trunk (LGST) was kept in the fixative solution (approximately 2 h). The tissue nervous was cryoprotected in sucrose solution of increased concentration (10, 20 and 30% sucrose in phosphate buffer 1%). The nervous tissue was stored for 24–48 h in sucrose buffer solution at 4 °C, embedded in Tissue-Tek medium for frozen tissue specimens (Sakura Finetek USA, Torrence, CA) and cryostat sectioned (Micron HM 505 N Cryostat, Walldorf, Gemany) at 20 μm. The sections were mounted on poly-L-lysine (Slide adhesive solution, Sigma-Aldrich, St, Louis, MO, USA) and placed onto clean microscope glass slides.

The sections were analyzed with a fluorescence microscope (Olympus BX 41, Olympus Corporation, Tokio, Japan). A positive neuron to TB was seen through the ultraviolet light (340–380 nm excitation filters). The raw number of labeled neurons was adjusted using the formula for Abercrombie’s correction factor. The sections were photographed with a digital camera (Evolutions VF, Media Cybernetics, Canada). Digital photomicrographs were saved as tiff files, and the images were analyzed and measured using Image-Pro Plus 6 (Version 6.3 for Windows, Media Cybernetics, Bethesda, MD, USA) with automatic adjustment of brightness with the purpose to prevent the auto-fluorescence by a fixative solution. The results of the number of TB labeled neurons in the SMG and LGST were presented as mean ± standard error of the mean (SEM) for the eight rats. The area of the soma was presented as mean ± SEM of the 10 neurons per rat by tracing the outline of the labeled cell body and calculating the enclosed area.

## Results

### Anatomical description of the ovarian plexus nerve

**Fig. 1:**
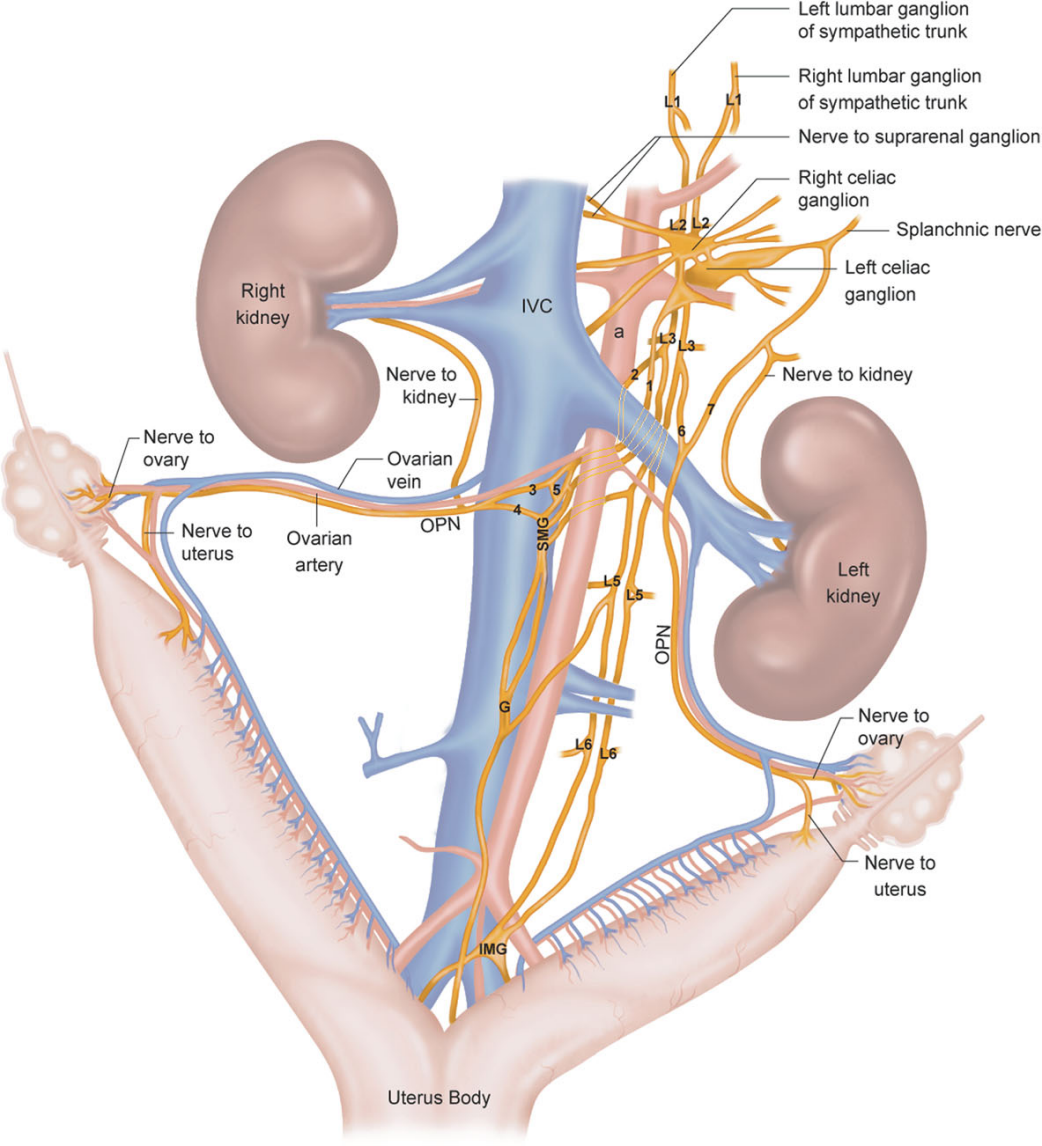
Schematic showing the origin and distribution of the ovarian plexus nerve, as well as the localization of the superior mesenteric ganglion of the female rat. OPN, Ovarian plexus nerve; a, Aorta; IVC, Inferior vena cava; SMG, Superior mesenteric ganglion; G, Ganglion; IMG, Inferior mesenteric ganglion; L (1,2,3,4,5,6) Lumbar ganglion of sympathetic trunk. 1: Celiac branch, 2: Lumbar branch, 3: Anastomotic branch, 4: Superior mesenteric branch, 5: Mesenteric plexus, 6: Anastomotic branch of the lumbar ganglion of sympathetic trunk, 7: Anastomotic branch of the splanchnic nerve

The OPN is a bilateral nerve and it has a length of 2.5 to 3 cm from the ovary to the first prevertebral ganglion. The right and left OPN runs adjacently and along the ovarian artery and bifurcates for approximately 3 mm before it gets to the ovary, the other branch goes to the uterus (Figs. 1 and 5).

**Fig. 2:**
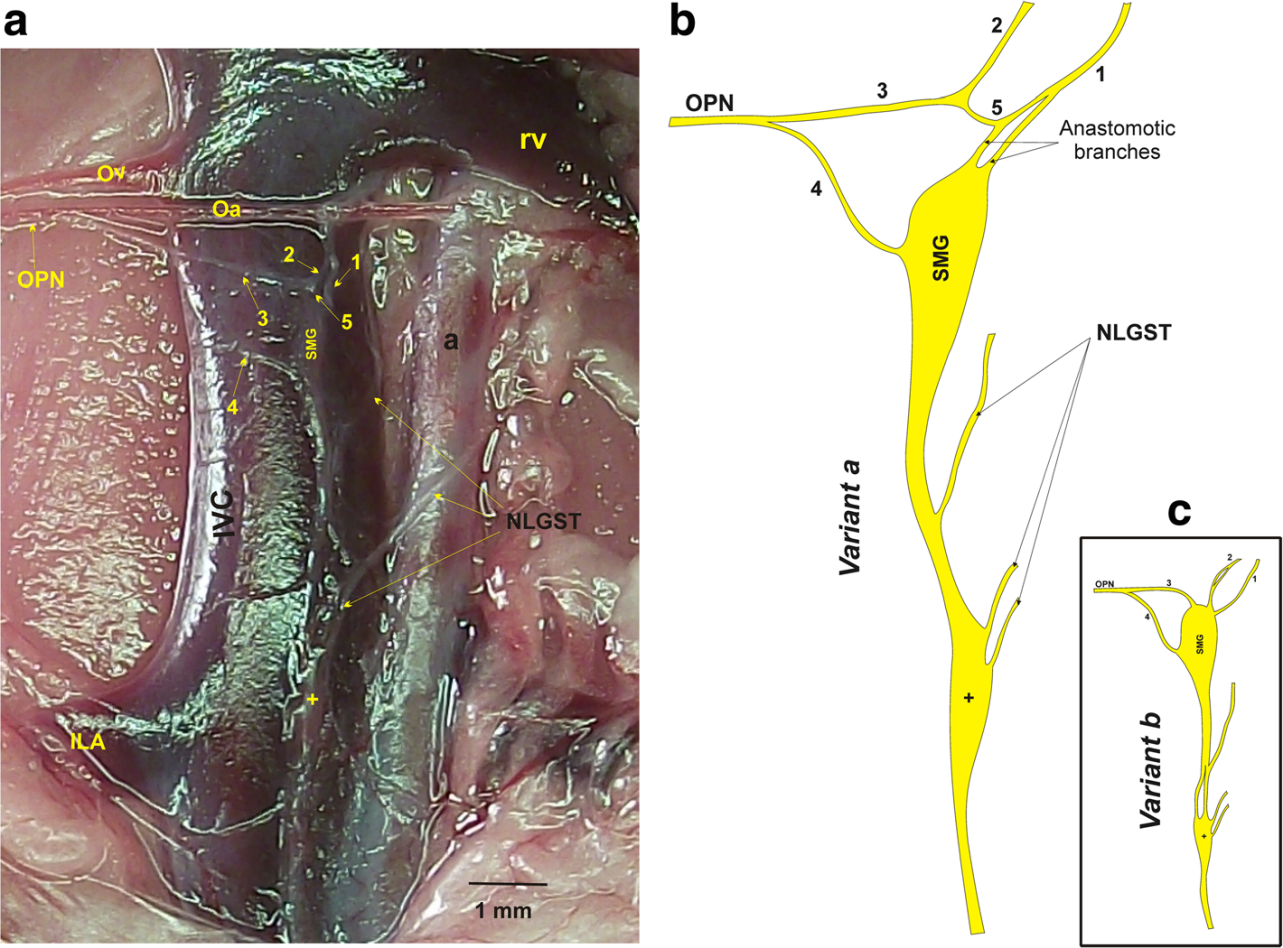
Digital images and schematic representation of the SMG attached the inferior vena cava and distribution of the branches of the female rat. **a** Photomicrograph showing the location of the SMG. 1: Celiac branch, 2: Lumbar branch, 3: Anastomotic branch, 4: Superior mesenteric branch, 5: Mesenteric plexus. 3 and 4 Branches converge to form the OPN; IVC, Inferior vena cava; Ov, Ovarian vein; rv, Renal vein; Oa, Ovarian artery; NLGST, Nerves to lumbar ganglion of sympathetic trunk; Small ganglion (+). **b** and **c** Variations in the gross anatomy of the SMG attached the inferior vena cava. Drawing: One organization was found in 63% of case (**b**) and the other in 37% of cases (**c**). The numbers are related to the nomenclature of the nerves. One nerve coming from the left celiac ganglion; two nerves coming from the lumbar ganglion of sympathetic truck; the three branches arise to the anastomotic branches (**b**); the three branches (**c**) emerge directly from the SMG like the four branch

The right OPN (R-OPN) has a dual origin: one branch arises from the SMG and the second one from the mesenteric plexus (Fig. 1, number 5). The postganglionic fibers of the R-OPN show two arrangements in relation to its origin (Fig. 2a, b). In 63% of the rats, one plexus under the renal vein (Fig. 2a) was found ventrolaterally attached to the inferior vena cava. This plexus consists of two nerves: lumbar (Fig. 2, number 2) and celiac branch (Fig. 2, number 1). The first one arises from the LGST, while the second one from the celiac ganglion (CG). The lumbar branch joins the anastomotic one (Fig. 2, number 3) whereas the celiac branch follows two paths: the anastomoses to the SMG and the other one joins the lumbar branch (Fig. 2, number 2). On the other hand, in 37% of the rats, the SMG and the anastomotic branches that originate the R-OPN come directly from the SMG. The lumbar (Fig. 2b, number 2) and celiac branch (Fig. 2b, number 1) go directly to the cranial part of the SMG (Fig. 2c).

The left OPN (L-OPN) postganglionic fibers originate from the anastomotic branch of the LGST (Fig. 1, number 6), and the anastomotic branch from the splanchnic nerve (ABSN) (Fig. 1, number 7). Before the L-OPN innervates the ovary for approximately 3 mm before it gets to the ovary, the other branch goes to the uterus (Fig. 1).

### Gross anatomy of the superior mesenteric ganglion

The SMG is the only ganglion located in the right side, and it is attached to the inferior vena cava. Three nerves arise from the SMG: two cranial that anastomose in the celiac, and the lumbar branch. The last two join the anastomotic branch and they form right OPN. The intermesenteric nerve arises caudally from the SMG, and it goes through the ventrolateral wall of the inferior vena cava and joins a small ganglion located in the inferior vena cava. The fibers of this ganglion reach the inferior mesenteric ganglion (IMG; Fig. 2a, b).

### Histologic features of the superior mesenteric ganglion

**Fig. 3:**
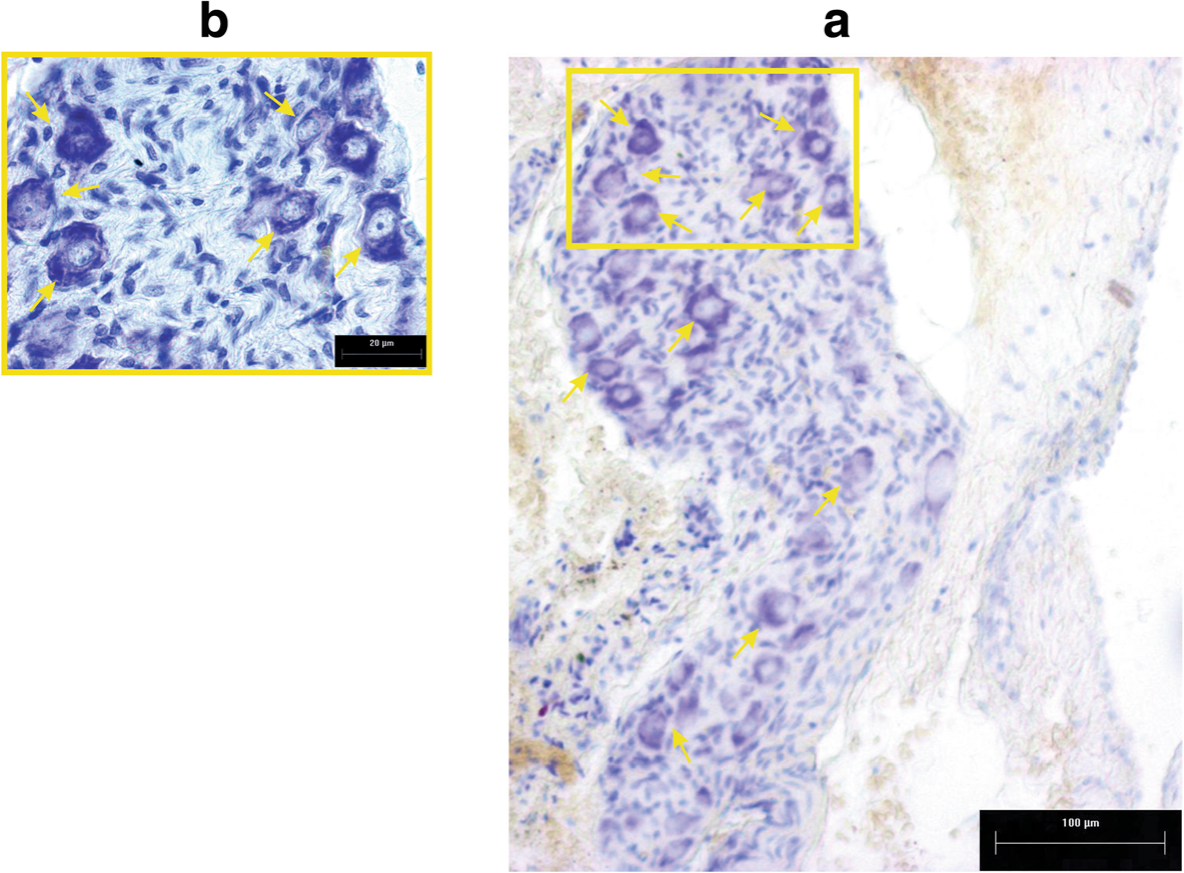
Photomicrograph longitudinal sections of SMG of rat stained with Nissl stain. **a** Nissl positive neurons distributed in SMG. **b** Neurons at higher magnifications. Note oval shape. Magnification 100×; bar 5 μm

The SMG has a spindle shape (area 3824 μm^2^), and it contains 177 ± 26 neurons (numbers adjusted using Abercrombie’s formula) (Fig. 3a). It was observed that the shape of the SMG neurons was oval. The majority of the nuclei were localized in the center of the perikarya (Fig. 3b). The area of the majority of the neurons was 84 ± 4 μm^2^.

### Sympathetic postganglionic neurons

**Fig. 4:**
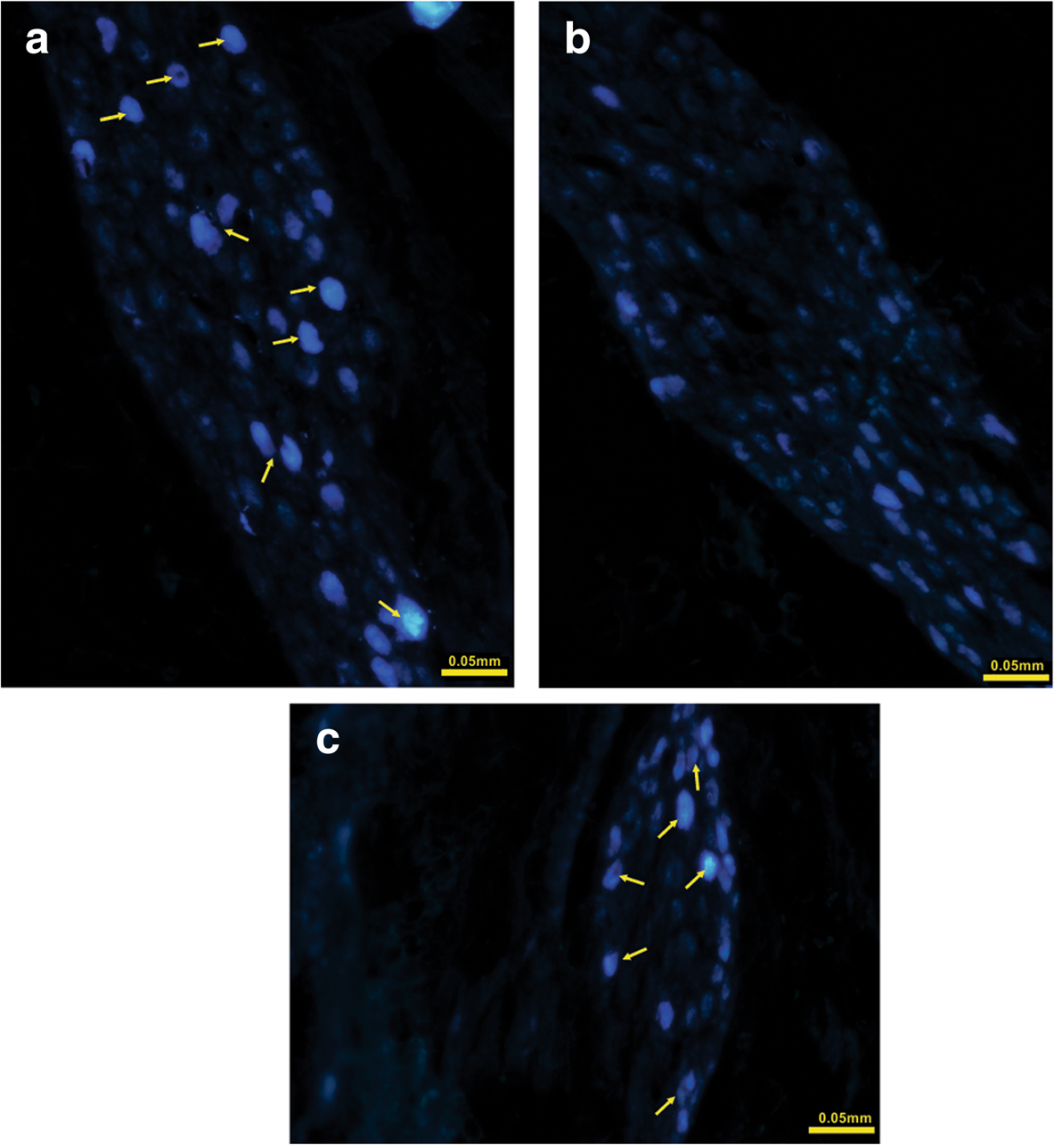
Representative photomicrographs of True Blue (TB) labeled postganglionic neurons of the ovary. **a** Labeled neurons (*white arrow*) in the SMG attached the inferior vena cava when TB was injected into right-ovarian bursa were observed. **b** Non-labeled neurons in the SMG when TB was injected into left-ovarian bursa were found. **c** Labeled neurons (*white arrow*) in the first LGST when TB was injected into left-ovarian bursa were found

When the TB tracer was injected into the right ovary, labeled postganglionic neurons were observed in the SMG (30 ± 8 neurons; Fig. 4a). In contrast, when the TB was injected into the left ovary, labeled postganglionic neurons were not observed in the SMG (Fig. 4b); however, labeled postganglionic neurons were observed in the first LGST (58 ± 17.4 neurons; Fig. 4c).

## Discussion

The results of the present study show that the postganglionic axons of the OPN that innervate the right and left ovary show different pathways. The right OPN fibers originate from the SMG and the LGST, while the left OPN fiber arises from the LGST. A similar description of the postganglionic neurons source was made by Baljet and Druker [2]: “the ovarian nerves are derived from the celiac plexus, the intermesenteric plexus and the upper lumbar splanchnic nerves”; however, this description does not show the laterality of the ovarian innervation.

On the other hand, different authors [2, 4, 6, 14, 16] describe that the postganglionic fibers of the OPN emerge from the celiac ganglion, which suggests that autonomic innervation of the ovaries shows an apparent asymmetry in the neural connections with the prevertebral ganglia. This provides evidences of the multisynaptic neural pathway between the ovary and the peripheral nervous system.

The SON plays an important role in the communication process between the ovaries and prevertebral ganglion [4, 8]. Previous reports showed that the presence of the neural information that goes from the ovary to the ganglion is ipsilateral [19]. In a 24-day-old rat, sectioning of the left SON results in a decrease of TB stained neurons in the left CSMG, though not completely eliminating the presence of stained cells. It is proposed that the neural connection between the left CSMG and the left ovary is carried by the OPN and the SON. The neural connection between the right CSMG and the right ovary is carried only by the OPN, because the section of the right SON does not modify the number of labeled cells [20]. The results of the present study agree with the latter in that the pathway of communication from the right ovary to the prevertebral ganglion through the right OPN is more complex. That is, it is a neural network which is part of the SMG.

**Fig. 5:**
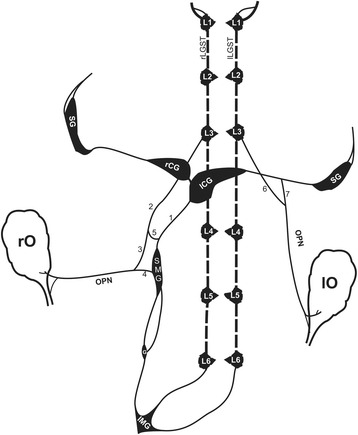
Representation of the main groups of sympathetic ganglia related to the ovary. The numbers correspond to the nomenclature of the branches. 1, Celiac branch; 2, Right lumbar branch; 3, Anastomotic branch; 4, SGM branch; 5, Mesenteric plexus; 6, Anastomotic branch of the lumbar ganglion; 7, Anastomotic branch of the splanchnic nerve; L (1,2,3,4,5,6) lumbar ganglion of sympathetic trunk; G, Ganglion; IMG, Inferior mesenteric ganglion

These results depict that the OPN runs contiguously to the ovarian artery, and 3 mm before arriving to the gonad, the OPN bifurcates into a branch to the upper part of the uterus. We did not observe the existence of a branch innervating the oviduct as previously described by Nance et al. [11]. We observed the presence of fibers that originate from the SMG and arrive to the right ovary and the upper part of the uterus through the OPN meaning that the SMG is part of the prevertebral ganglionic structures that regulate the right ovary and uterus functions. It can be concluded that the SMG is the ganglia attached to the ventral wall of the inferior vena cava, and it is connected to the celiac ganglia, the LGST and connects caudally with IMG (Fig. 5).

In previous studies, it was shown that when neuronal tracer was injected into the right ovary, labeled neurons were observed in a small ganglion located in the angle between the aorta and the renal arteries [19].

Similarly, McNeill and Burden [15] observed labelled ovarian postganglionic neurons distributed in the smaller ganglia (caudal preaortic ganglia) located in the origin of the renal and ovarian arteries. Moreover, the gross anatomical analysis showed the presence one or two smaller ganglia that connects the right celiac with the left celiac ganglia. Berthoud and Powley [12] showed a ganglionated intermesenteric plexus that connects the superior with the inferior mesenteric ganglia on the ventral surface of the aorta.

Hamer and Santer [17] showed the presence of the ovarian ganglia located ventrally and caudally where the ovarian arteries originate located around the aorta. The difference between those results with the present work lies in that we show that the ganglia is attached to the inferior vena cava, and it is caudally located in the ovarian artery.

Regarding the presence of paired celiac ganglia, our results agree with those described by other authors [2, 12, 17].

Baljet and Drukker [2] describe the caudal part of the left celiac ganglion is the SMG. Nevertheless, in the present study, the segment in the caudal part of the left celiac ganglion was observed below the superior mesenteric artery and is part of the same ganglion. Therefore, the SMG is not a segment of the left CG. It should be noted that the SMG has been identified in humans [21] and cats [22], but never in rats.

## Conclusions

Our findings show that in female rats, the right OPN originates from the branch of the SMG and from the anastomotic branch and emerges from the SMG, CG and the first LGST. The left OPN postganglionic fiber originates from the first LGST and ABSN. The SMG is unilateral and contains postganglionic neuros innervating the right ovary and uterus. Due to the fact that most of the basic physiology of this research was carried out in the ovaries of the rats, it is important to consider these results in applied research and ovarian pathologies, which demonstrate that in addition to the celiac ganglion, other ganglia facilitate the communication between the ovaries and the central nervous system.

## Abbreviations

ABSN: Anastomotic branch from the splanchnic nerve
CG: Celiac ganglion
CSMG: Celiac-superior mesenteric ganglia
DRG: Dorsal root ganglia
G: Ganglion
IMG: Inferior mesenteric ganglion
LGST: Lumbar ganglion of the sympathetic trunk
L-OPN: Left OPN
OPN: Ovarian plexus nerve
R-OPN: Right OPN
SG: Suprarenal ganglion
SMG: Superior mesenteric ganglion
TB: True Blue
